# Supplementary material for: Chromothripsis during telomere crisis is independent of NHEJ, and consistent with a replicative origin
Source: Genome Res. 2019 May;29(5):737–49. doi: 10.1101/gr.240705.118 (PMC6499312; doi:10.1101/gr.240705.118)
Supplement: Supplemental Material [file supp_gr.240705.118_Supplemental_file_1.zip › contigs/annotated_contigs/DB53/contig.2.DB53_length_548_mean_cov_9.85401459854.docx]

**DB53_length_548_mean_cov_9.85401459854**

```
AGAATCCAAACTAAAATTAACCATAAGTAGCTCTACATGGTAAGACCAAAGAGTACAAGGAATGGTGTGCAGGGACAAAAGATACTGCA
 >chr6:37214775-37215033 - E=1e-143
GCAGGGGTCTATCATAGTAGACTTCTAAGGCCATATAAGTCTTATGTTACCTTTTCCTCTAGGCAATGGGACATTCATTGCCAGTTTAA

GCTGGGACATGACATGACCAGATCTGCATTGTAGAAGGACCACTCTAGTCACATATCTATTAAATGGCTTCTGCTACAC|T|GTTTATG
                                                                                >chr6:3
TGATGTTATTGTGACAATATACGGCCAACACACATATGATTCTCCAGATTTCTCCACAAGTCTATGAGCCATGGTTTTTCTGCTTAAAT
7212179-37212470 - E=2e-163
CAGCCAAGAGTTGGCACTTGTTGCTTGCTCTGACTTACACAGAGAAAGACTGAAAGTAAATTAGAGAGAAACATGGGGTCAAGGGAGAA

GTTGAGGTAGACGAGACTTCAACATGTTTATAGGCCAAGGATTGGAGGCAGTTGTATTTGTTAGGCTATAAGTGTAGCTGGAAAATAAT

AGTGCTTTAGACACAA
```
